# Mobile Application for Perceived Stress and Self‐Efficacy Management of Caregivers of Elderly Patients With Parkinson′s Disease

**DOI:** 10.1155/ijta/3978713

**Published:** 2026-03-04

**Authors:** Hossein Sanaei, Najmeh Valizadeh Zare, Tahereh Sadeghi, Mohsen Soltani Sabi, Ali Shoaibi

**Affiliations:** ^1^ Nursing Department, Healthy Ageing Research Center, Neyshabur University of Medical Sciences, Neyshabur, Iran, neyshabur.ac.ir; ^2^ Geriatric Nursing Department, Faculty of Nursing and Midwifery, Nursing and Midwifery Care Research Center, Mashhad University of Medical Sciences, Mashhad, Iran, mums.ac.ir; ^3^ Pediatric Nursing Department, Faculty of Nursing and Midwifery, Nursing and Midwifery Care Research Center, Mashhad University of Medical Sciences, Mashhad, Iran, mums.ac.ir; ^4^ Department of Neurology, Faculty of Medicine, Mashhad University of Medical Sciences, Mashhad, Iran, mums.ac.ir

**Keywords:** caregivers, mobile application, Parkinson′s disease, perceived stress, self-efficacy

## Abstract

**Introduction:**

Parkinson′s disease (PD) places a substantial burden on caregivers, affecting their quality of life and potentially compromising patient care. Mobile health (mHealth) interventions may help reduce these challenges. This study was aimed at evaluating the effect of a mobile application on perceived stress and self‐efficacy among caregivers of older adults with PD.

**Method:**

This randomized controlled clinical trial was conducted with 80 caregivers recruited from the Neurology Clinic of Qaim Hospital, Iran. Participants in the intervention group received access to a PD management mobile application along with face‐to‐face training, while the control group received only face‐to‐face training at the clinic. Both groups completed the Cohen Perceived Stress Inventory and the Caregiver Self‐Efficacy Scale at baseline, immediately after the intervention, and 1 month later.

**Results:**

Immediately after the intervention, the intervention group demonstrated significantly lower perceived stress compared to the control group (*p* = 0.018). However, this difference was not sustained at the 1‐month follow‐up (*p* = 0.115). Within‐group analyses showed no significant change in stress levels over time (*p* > 0.05). Self‐efficacy scores improved in the intervention group, particularly in the domains of “gathering information about treatment” (*p* = 0.031) and “completing household tasks” (*p* = 0.041).

**Conclusion:**

The mobile application improved caregivers′ self‐efficacy and temporarily reduced perceived stress, suggesting its potential as a supportive tool for individuals caring for older adults with PD. Integrating mHealth solutions may enhance caregiver well‐being and contribute to better caregiving outcomes.

## 1. Introduction

Parkinson′s disease (PD) is the most common neurodegenerative disorder, affecting nearly 1% of individuals over the age of 50 [1, 2]. Caring for older adults with chronic neurological conditions places substantial emotional, physical, financial, and social burdens on family caregivers, often diminishing their quality of life and compromising the care they provide [3–5]. Despite increased attention to the positive aspects of caregiving, caregivers frequently experience depression, anxiety, stress, and decreased life satisfaction and are sometimes referred to as “hidden patients” due to their vulnerability to mental health problems [6–8].

Research shows that caregivers of individuals with neurological diseases face considerable stress and challenges related to both caregiving tasks and the emotional adjustment to caregiving responsibilities [9, 10]. Traditional support and training programs typically require face‐to‐face interactions and the physical presence of both caregivers and trained professionals, limiting accessibility and scalability [11, 12].

Self‐efficacy plays a central role in enabling caregivers to manage symptoms effectively. According to Bandura′s theory, self‐efficacy reflects confidence in one′s ability to perform required behaviors and is an important determinant of coping and performance in caregiving tasks [13, 14]. Several interventions including progressive muscle relaxation, family‐centered empowerment programs, collaborative training, and peer support have shown benefits in improving caregiver outcomes in various chronic conditions. However, evidence remains inconsistent, and these studies do not specifically address mobile application–based interventions for caregivers [15–17]. This highlights a significant gap in research regarding the role of mobile health (mHealth) tools in reducing perceived stress and enhancing caregiver self‐efficacy.

In the post COVID‐19 era, digital health technologies have gained prominence as cost‐effective, time‐efficient alternatives to traditional face‐to‐face training. Studies have demonstrated the utility of mobile applications for monitoring movement, speech, and functional status in patients with PD [18–21]. Furthermore, mobile applications designed for caregivers of older adults have shown promise in improving communication with healthcare teams and supporting home‐based care[22]. While mHealth interventions have demonstrated benefits in the management of chronic diseases such as diabetes and heart failure, evidence specific to caregivers of individuals with PD is limited and at times inconclusive [23, 24].

Given the growing need for accessible, scalable, and user‐friendly solutions for caregiver support, mobile applications may serve as an effective tool for delivering education, enhancing skills, and promoting psychological well‐being. Therefore, this study was aimed at evaluating the effect of a disease management mobile application on perceived stress and self‐efficacy among caregivers of older adults with PD.

## 2. Study Hypotheses

It was hypothesized that caregivers who used the disease management mobile application would demonstrate significantly lower levels of perceived stress and significantly higher levels of self‐efficacy compared to those receiving routine care.

## 3. Method

This research is designed as a two‐group randomized controlled clinical trial, registered under the code IRCT20210530051443N1. All procedures adhered to the ethical guidelines established by Mashhad University of Medical Sciences (IR.MUMS.NURSE.REC.1400.003). Participants provided informed consent after being assured that their data would be kept confidential. The study population consisted of caregivers of elderly patients with PD who are referred to the neurology clinic of Qaim Hospital in Iran. These centers were equipped with appropriate resources and staffed by qualified professionals to facilitate the research.

Inclusion criteria for elderly caregivers included (1) a minimum duration of caregiving for an elderly individual with PD, (2) ownership of a smartphone with an Android operating system, (3) age between 18 and 60 years, (4) literacy in reading and writing, (5) no history of mental disorders, and (6) no current use of antianxiety or sedative drugs. Exclusion criteria included the occurrence of any acute debilitating disease during the study and a lack of willingness to continue participation.

### 3.1. Patients

**Figure 1:**
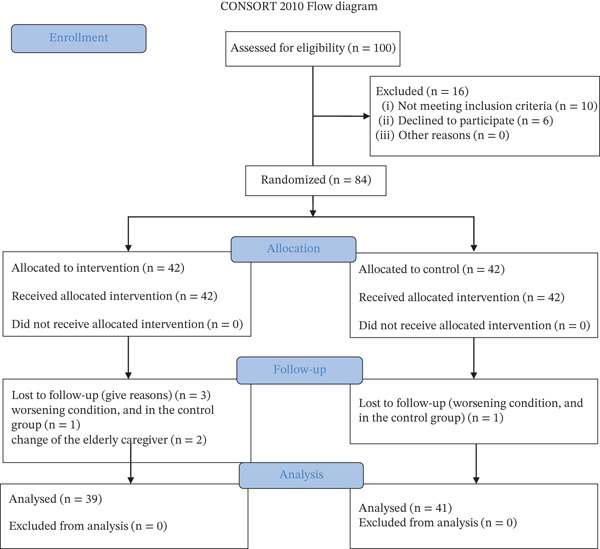
Consort diagram.

The sample size was calculated based on the self‐efficacy variable. With an effect size of 0.69, a Type 1 error rate of 0.05, and a desired statistical power of 0.90, the final sample size was determined to be 74 participants. To account for an anticipated dropout rate of 30%, a total of 100 people were selected through convenience sampling. After introducing the research and obtaining informed consent, random allocation was performed using a block randomization method (10 blocks of 4 participants). Blocks were randomly generated using the GraphPad website. After an initial review, 16 caregivers were excluded from the study due to noncompliance with the inclusion criteria. Finally, 84 participants who met the inclusion criteria were randomly assigned to two equal groups: intervention and control. In the intervention group, one participant was excluded due to the patient′s worsening condition, while in the control group, three participants were excluded: one due to the patient′s deteriorating condition and two due to a change in the elderly caregiver. Figure 1 illustrates the flow of participants throughout the study, as recommended by the CONSORT guidelines.

### 3.2. Procedure

This study was conducted in two phases.

#### 3.2.1. Phase 1: Content Preparation and Validation

The educational content for the application was compiled based on a review of recent literature, covering PD symptoms, complications, management strategies, nutrition, exercise, and medications. Content was reviewed by neurologists and 10 field experts to ensure accuracy and validity. The Mobile App Rating Scale (MARS) was used to assess application quality and user satisfaction, with favorable scores reported in information quality and engagement (see File S1 for detailed content, validation, and testing procedures).

This mobile application is designed as a comprehensive, fully Persian‐language platform for managing PD, addressing the needs of patients and their families through clear, accessible, and nontechnical communication. Its content is intentionally written in simple, everyday language so that any individual regardless of medical background can use it with ease. The educational section includes more than 100 high‐quality and easily understandable images, providing explanations of the brain pathways affected in PD and the mechanisms of action of commonly used medications such as levodopa and other drugs, as well as visual demonstrations of key motor symptoms including hand tremor, bradykinesia, rigidity, gait freezing, balance difficulties, and falls. Nonmotor symptoms such as constipation, sleep disturbances, depression, and reduced sense of smell are also explained in a friendly and conversational tone, similar to speaking with a close companion. The educational materials are concise and user‐friendly, supported by audio narration in calm, natural Persian, allowing users to listen to the content when reading is difficult or tiring. Simple three‐dimensional animations illustrate how the brain functions and how medications assist in managing symptoms. In addition, multiple short, precise videos demonstrate Parkinson‐specific exercises, including strength and balance training programs, with slow, easy‐to‐follow movements and Persian explanations. The medication reminder system enables users to easily enter drug names, dosages, and exact administration times. It provides repeated sound and vibration alerts and displays a clear color‐coded report showing the percentage of doses taken on time. The system also offers reminders for daily exercise, hydration, sleep routines, and medical appointments. User accounts are protected through secure two‐step authentication, full encryption of personal information, and an option to grant limited access to a family member or caregiver. The “About Us” section introduces the medical professionals and specialists involved in the project with photos and concise descriptions, lists 50 up‐to‐date and credible references, and displays the date of the most recent content update.

#### 3.2.2. Phase 2: Implementation and Use

After ethical approval, 80 caregivers were randomly assigned to the intervention or control group. The intervention group received the mobile application along with face‐to‐face training and was instructed to engage with educational materials every 3 days. Participants could ask questions via the application or WhatsApp, and weekly follow‐up was conducted by the researcher. The control group received standard face‐to‐face training in the clinic. Data were collected using the Cohen′s Perceived Stress Inventory and the Caregiver Self‐Efficacy Scale at baseline, immediately postintervention, and 1 month later. After study completion, the application was provided to the control group.

File S1 contains detailed information about the application development process, including technical specifications, black‐box and white‐box testing, Monkey and JUnit tests, and full MARS questionnaire results.

The application incorporated a range of features designed to enhance user experience and educational efficacy. These included direct communication with the application administrator, a question‐and‐answer platform for interaction with the treatment team, access to up‐to‐date and reliable information, content categorization to minimize confusion, the ability to send notifications to users, and the integration of multimedia elements such as GIFs, audio, photographs, and text to facilitate better learning.

#### 3.2.3. Phase 2: The Implementation and Use of the Application

After the acquisition of ethical approval and necessary permits, qualified caregivers were identified and randomly assigned to either the intervention or control group. The intervention group used the PD management application, receiving hands‐on training during face‐to‐face sessions, during which the application was installed on their mobile phones. Conversely, the control group received similar face‐to‐face training in a clinical setting.

The intervention group initially attended a session aimed at teaching them how to use the application, integrated into the faculty′s educational offerings. After the training, the caregivers were instructed to engage with at least one section of the educational materials every 3 days. Upon completing a section, the caregivers could pose questions to the researcher via the application or WhatsApp. Throughout the duration of the study, the researcher maintained weekly contact with participants to discuss their use of the application and any issues that arose. To prevent information exchange between the two groups, user accounts were created for each participant in the intervention group, restricting access to others outside the intervention team. Data collection utilized the Cohen′s Perceived Stress Questionnaire and the Caregiver Self‐Efficacy Scale. These instruments were administered in three phases: before the intervention, immediately after it, and 1 month postintervention. The control group received training from a physician or nurse at the clinic. At the end of the study, the application was made available to the control group.

### 3.3. Evaluation

The perceived stress and caregiver self‐efficacy questionnaires served as the primary evaluation tools for this study. The validity of the self‐efficacy questionnaire was confirmed through consultations with 10 experts in nursing and midwifery. Developed by Zhang et al., the scoring for this questionnaire ranges from 0 to 3500, with a mean baseline score of 1750. Higher scores indicate greater self‐efficacy, while lower scores reflect diminished self‐efficacy ([14] #11). The translation of the questionnaire was carried out using a forward and backward translation technique, receiving final approval from the main developer. The instrument exhibited sufficient reliability, with Cronbach′s alpha coefficients ranging from 0.80 to.89 points for the entire scale.

The validity of the Perceived Stress Questionnaire was also established through expert consultation, yielding a correlation coefficient of 0.76 ([25] #19; [25] #19). This questionnaire consists of 14 questions scored on a 5‐point Likert scale, where responses range from “*never*” to “*always*,” with scores assigned from 0 to 4. Specific questions are scored in reverse (4, 5, 6, 7, 9, 10, and 13). A total score below 28 indicates low perceived stress, while a score of 28 or above categorizes the respondent as experiencing high perceived stress.

### 3.4. Statistical Analysis

Data analysis was conducted using SPSS Software Version 21. Descriptive statistics, including frequency distribution, mean, and standard deviation, were used to describe the research sample. Analytical statistics were employed to examine the homogeneity of the two groups and to achieve the research objectives. The Shapiro–Wilk test was conducted to determine the normality of the quantitative variables. The independent *t*‐test was used for assessing group homogeneity concerning quantitative confounding variables, assuming normal distribution, while the Mann–Whitney test was utilized for variables exhibiting nonnormal distribution. Ordinal variables were similarly analyzed using the Mann–Whitney test, and nominal variables were compared between the two groups using the chi‐square test.

To compare the mean scores of perceived stress and self‐efficacy, as well as their respective dimensions across the two groups, the independent *t*‐test was used for normally distributed data, whereas the Mann–Whitney test was applied for nonnormally distributed data. Repeated measures analysis of variance was employed to examine changes in perceived stress scores and self‐efficacy within the groups in three consecutive measurements. All tests used a 95% confidence interval and a significance level of 0.05.

## 4. Results

A total of 80 participants were included in the study, comprising 41 individuals in the intervention group and 39 in the control group. No significant differences were observed between groups regarding demographic factors (*p* > 0.05), duration of PD (*p* = 0.074), patient age (*p* = 0.969), or caregiver age (*p* = 0.937) (Table 1). These findings suggest that the randomization process was effective. Moreover, there was no significant difference between the two groups in their initial perception of stress as detrimental (*p* = 0.741) (Table 2).

**Table 1:** Perceived stress and self‐efficacy scores.

Variable	Group	Baseline	Postintervention	1‐month follow‐up	*p* value (time group)
Perceived stress, mean (SD)	Intervention	28.43 (4.48)	24.02 (5.31)	27.75 (4.83)	0.009
	Control	27.05 (4.44)	27.68 (4.04)	30.48 (3.00)	0.234
	*p* value (between groups)	0.741	**0.018**	0.115	
Caregiver self‐efficacy, mean (SD)	Intervention	172.58 (21.82)	198.39 (53.95)	207.69 (56.24)	0.001
	Control	170.02 (22.02)	172.50 (21.97)	185.83 (29.97)	0.002
	*p* value (between groups)	0.696	**0.013**	**0.040**	

*Note:* Bold values indicate significant within‐group changes from baseline (*p* < 0.05). *p* values for between‐group comparisons were obtained from independent samples *t*‐tests at each time point. *p* values for within‐group comparisons and interaction effects were derived from repeated measures ANOVA.

Abbreviation: SD, standard deviation.

**Table 2:** Participant characteristics.

Variable	Intervention (*n* = 41)	Control (*n* = 39)	*p* value
Patient age (years), mean (SD)	72.05 (7.18)	72.21 (8.18)	0.969
Caregiver age (years), mean (SD)	36.72 (10.46)	36.49 (11.08)	0.937
Duration of PD (years), mean (SD)	1.68 (0.05)	2.13 (1.13)	0.074
Gender (patient), *n* (%)			
Male	30 (73.2)	31 (79.5)	0.507
Female	11 (26.8)	8 (20.5)	
Relationship to patient, *n* (%)			
Child	19 (46.3)	25 (64.1)	0.162
Spouse	10 (24.4)	2 (5.1)	
Other	12 (29.3)	12 (30.8)	

*Note:* *p* values are from independent samples *t*‐tests (for normally distributed data) or Mann–Whitney *U* tests (for nonnormally distributed data).

Abbreviation: SD, standard deviation.

In the analysis of the immediate postintervention effects, it was observed that the perceived stress level in the intervention group was significantly lower than that in the control group (*p* = 0.018). However, at the 1‐month follow‐up, no significant difference was found between the groups (*p* = 0.115). Within‐group analyses revealed that perceived stress levels did not change significantly over time (*p* > 0.05).

At baseline, the groups were also comparable in terms of self‐efficacy levels (*p* = 0.696). The intervention group showed significantly higher self‐efficacy than the control group immediately after the intervention (*p* = 0.013) and at the 1‐month follow‐up (*p* = 0.040). A priori analyses of within‐group changes in self‐efficacy over time revealed a statistically significant increase in both the intervention group (*p* = 0.009) and the control group.

Follow‐up results indicated that the intervention group was significantly more likely to feel confident in their ability to respond to problematic behaviors (*p* < 0.001) and reported having received sufficient support during this period (*p* < 0.001). Analysis of the caregiver response to C‐RCSES subscales further indicated that within‐group analyses over time showed significant improvements in the intervention group in “gathering information about treatment” (*p* = 0.031) and “completing household tasks” (*p* = 0.041) (Table 1).

## 5. Discussion

The results of the study showed that the use of the Parkinson′s assistant educational app significantly reduced perceived stress and increased self‐efficacy in caregivers of elderly people with PD. Immediately after the intervention phase, perceived stress levels in the intervention group were significantly reduced compared to those in the control group. This result is consistent with the study by Long et al. (2020), which reported that family caregivers of dementia patients experienced significant improvements in self‐efficacy, reductions in depressive symptoms, and decreased stress through these interventions that included mobile applications [26]. Similarly, Jin et al. (2022) found that a therapeutic imagery smartphone app significantly improved coping skills and reduced stress levels among dementia caregivers [27]. These findings suggest that concurrent health interventions have the potential to provide readily accessible support and personalized strategies to reduce the negative effects of caregiver burden. Our study found that the reductions in perceived stress were not sustained at 1‐month follow‐up. In contrast, some studies have shown that caregiver stress levels continue to decrease after concurrent health interventions ([28]; [26]). This discrepancy may be attributed to the relatively short intervention period of 1 month; as other studies suggest, longer interventions may be needed to achieve lasting changes in coping strategies and stress responses [29]. Additionally, confounding factors such as rising healthcare costs and medication shortages for patients with PD may contribute to increased caregiver stress levels and affect the effectiveness of follow‐up care. This reflects the complex realities faced by caregivers of patients with chronic diseases such as PD, where psychological well‐being is influenced by factors beyond patient care responsibilities. Further research is needed to explore how stressors such as financial pressures and difficulties in accessing medications affect the long‐term effectiveness of health‐related interventions. A study by Durst et al. also showed that health information technology may not effectively reduce stress for pregnant women and their caregivers in the long term. These results suggest that while mobile education may serve as an effective intervention for certain care outcomes, its effect on reducing stress related to patient illness may vary based on the specific caregiving needs. Sustainable reductions in perceived stress may depend on developing interventions tailored to address the unique stressors faced by specific caregiver groups, such as those caring for people with PD [30]. Our research found that caregivers in the intervention group had significantly higher levels of self‐efficacy than those in the control group, both immediately after the intervention and at the 1‐month follow‐up. These findings are consistent with a systematic review conducted by Shumenghui Zhai et al. (2023) [31], which examined 40 studies across 34 journals and showed that the use of digital health tools leads to improved psychological outcomes, self‐efficacy, and enhanced problem management in caregivers of patients with chronic diseases [31]. Easy access to treatment plans can increase self‐efficacy and reduce stress due to a lack of treatment knowledge. This finding aligns with a large body of research demonstrating that accompanying health interventions increase caregivers′ self‐efficacy in managing chronic illnesses [32, 33]. For example, Akbari et al. (2021) found that caregivers of cancer patients experienced significant improvements in self‐efficacy and sense of coherence after participating in an online learning program [33]. Similarly, Khachian et al. (2023) reported that a self‐management training program based on a mobile application significantly improved the quality of life for people with PD, which may indirectly enhance caregiver self‐efficacy by reducing caregiving demands [34]. Collectively, these studies, including our own, show that health interventions can empower caregivers by providing them with the necessary information, skills, and support to successfully manage their caregiving responsibilities. The educational materials, self‐management features, and communication tools offered by these applications likely contribute to enhancing caregivers′ sense of control and confidence.

It is noteworthy that Park et al. (2022) [35] also found that concurrent health interventions positively impacted self‐efficacy; however, their study focused on the self‐management of patients with PD rather than caregivers. This distinction highlights the need for further research, especially in the area of supplementary health interventions aimed at enhancing caregiver self‐efficacy in the context of PD. In addition, our study identified significant improvements in specific self‐efficacy subscales, including “managing household chores,” “dealing with problem behaviors,” “receiving support,” and “gathering information about treatment.” These results are consistent with those reported by Khachian et al. (2023) [34], suggesting that the app′s capacity to increase caregivers′ confidence and competence in communication with healthcare providers, addressing challenging behaviors, and providing reliable information may have a direct and positive impact on their caregiving experience.

## 6. Conclusion

The results provide evidence for the potential of a smartphone application to aid in the management of PD by reducing perceived stress and increasing self‐efficacy in caregivers of older patients with PD. While the reduction in perceived stress was not sustained over the long term, the significant and lasting improvements in self‐efficacy highlight the importance of developing and implementing tailored mHealth interventions to support this population. Future research should focus on optimizing the design and content of such applications, investigating the effects of long‐term interventions, and addressing the multiple challenges faced by PD caregivers to maximize their well‐being and ability to provide optimal care.

## 7. Limitation

The psychological resilience and coping strategies of caregivers can vary widely, and the application may not adequately address these individual differences, which could result in variable effectiveness in stress management among users. Furthermore, caregivers work in different environments that provide varying levels of support, resulting in contextual influences that may affect their experiences of stress and self‐efficacy. The application may not fully account for these external factors, potentially limiting its overall effectiveness.

## Author Contributions

Hossein Sanaei and Najmeh Valizadeh Zare conceptualized the study, developed the draft proposal, and conducted the research. Tahereh Sadeghi, Mohsen Soltani Sabi, and Ali Shoaibi contributed to data collection and analysis. All authors participated in reviewing the draft manuscript.

## Funding

The study is supported by the Mashhad University of Medical Sciences, 10.13039/501100004748 (991439).

## Disclosure

All authors approved the final version.

## Ethics Statement

Ethical approval for this study was obtained from the Ethics Committee of Mashhad University of Medical Sciences (Ethics Code: IR.MUMS.NURSE.REC.1400.003). All procedures adhered to relevant guidelines and regulations, specifically the Declaration of Helsinki. Informed consent was obtained from all participants prior to their involvement in the study. Participants were clearly informed about the voluntary nature of their participation and their right to withdraw at any point. To maintain confidentiality and privacy, questionnaires were completed anonymously, using unique identifiers for each participant.

## Conflicts of Interest

The authors declare no conflicts of interest.

## Supporting information


**Supporting Information** The supporting information provides a detailed description of the development, validation, implementation, and evaluation phases of the Parkinson′s disease management application. It includes information on testing procedures, user feedback (MARS results), and the assessment instruments used in the study.

## Data Availability

The data supporting the findings of this study are accessible through the research deputy of Mashhad University of Medical Sciences; however, access is restricted due to the licensing agreement under which these data were utilized for this study. Data may be available from the corresponding author upon reasonable request and with the approval of the research deputy of Mashhad University of Medical Sciences.
