# Supplementary material for: Mobile Application for Perceived Stress and Self‐Efficacy Management of Caregivers of Elderly Patients With Parkinson′s Disease
Source: Int J Telemed Appl. 2026 Mar 4;2026:3978713. doi: 10.1155/ijta/3978713 (PMC12958131; doi:10.1155/ijta/3978713)
Supplement: Supplementary file 1 — Supporting Information The supporting information provides a detailed description of the development, validation, implementation, and evaluation phases of the Parkinson′s disease management application. It includes information on testing procedures, user feedback (MARS results), and the assessment instruments used in the study. [file IJTA-2026-3978713-s001.docx]

This study was designed and conducted in two phases:

**Phase 1: Content preparation and application validation**

In the first phase, the content for the application was compiled through a thorough review of the latest literature, including books and scientific articles. This content provided comprehensive information on Parkinson's disease, covering its symptoms, complications, management strategies, nutrition, movement care, and medications. Educational materials were presented to users in various formats, including videos, photographs, and text. The content of the application underwent a review process involving supervisors and neurologists, who provided corrective feedback that was subsequently integrated. To further validate the content, it was submitted to 10 experts in the field, and their recommendations were incorporated as well. After preparing the content and establishing its validity, the application was subject to two testing methodologies: black box testing and White box testing. These tests examined different aspects of the application such as its efficiency, usability, and user-friendliness. In the Monkey test, numerous automated screen touches were generated to examine the application's response under high input loads, while also recording and addressing any errors encountered. Additionally, the JUnit tool was used to conduct automated testing of the internal parts of the application. An application test was further employed to ensure the correct functionality of the application. To ensure the quality of the application, the Mobile App Rating Scale (MARS) questionnaire was used.The MARS questionnaire consists of two sections: App Quality Ratings and App Subjective Quality. The first section includes four domains with 19 questions, while the second section comprises four questions. All items on the MARS are rated on a 5-point scale ranging from “1. Inadequate” to “5. Excellent”. After the installation of the application, the MARS questionnaire was administered to 20 users, and their feedback regarding the various dimensions of the questionnaire was analyzed. (MARS) User satisfaction was found to be high according to the MARS ratings (n = 20). Users rated the application favorably, with particularly high scores in “information quality” and “engagement” achieving means of 28.95 out of 35 and 21.42 out of 25, respectively (table1), thereby indicating that the application was both appealing and provided authentic content.

**Table 1. Mobile App Rating Scale (MARS) Scores**

| **Mean and Standard Deviation of MARS Questionnaire Scores** | | | |
| --- | --- | --- | --- |
| **Domain** | **Minimum Score** | **Maximum Score** | **Mean and Standard Deviation** |
| Engagement | 17 | 25 | 21.42 (2.71) |
| Functionality | 14 | 20 | 17.14 (2.45) |
| Aesthetics | 11 | 15 | 13.33 (1.08) |
| Information Quality | 25 | 33 | 28.95 (3.08) |

**Note:** SD = Standard Deviation. Each item was rated on a 5-point Likert scale (1 = Inadequate to 5 = Excellent).

The application incorporated a range of features designed to enhance user experience and educational efficacy. These included direct communication with the application administrator, a question-and-answer platform for interaction with the treatment team, access to up-to-date and reliable information, content categorization to minimize confusion, the ability to send notifications to users, and the integration of multimedia elements such as GIFs, audio, photographs, and text to facilitate better learning.

**Phase 2: the implementation and use of the application**

After the acquisition of ethical approval and necessary permits, qualified caregivers were identified and randomly assigned to either the intervention or control group. The intervention group used the Parkinson's disease management application, receiving hands-on training during face-to-face sessions, during which the application was installed on their mobile phones. Conversely, the control group received similar face-to-face training in a clinical setting.

The intervention group initially attended a session aimed at teaching them how to use the application, integrated into the faculty's educational offerings. After the training, the caregivers were instructed to engage with at least one section of the educational materials every three days. Upon completing a section, the caregivers could pose questions to the researcher via the application or WhatsApp. Throughout the duration of the study, the researcher maintained weekly contact with participants to discuss their use of the application and any issues that arose. To prevent information exchange between the two groups, user accounts were created for each participant in the intervention group, restricting access to others outside the intervention team. Data collection utilized the Cohen's Perceived Stress Questionnaire and the Caregiver Self-Efficacy Scale. These instruments were administered in three phases: before the intervention, immediately after it, and one month post-intervention. The control group received training from a physician or nurse at the clinic. At the end of the study, the application was made available to the control group.

Evaluation

The perceived stress and caregiver self-efficacy questionnaires served as the primary evaluation tools for this study. The validity of the self-efficacy questionnaire was confirmed through consultations with ten experts in nursing and midwifery**.** Developed by Zhang et al., the scoring for this questionnaire ranges from 0 to 3500, with a mean baseline score of 1750. Higher scores indicate greater self-efficacy, while lower scores reflect diminished self-efficacy (Zhang, 2010). The translation of the questionnaire was carried out using a forward and backward translation technique, receiving final approval from the main developer. The instrument exhibited sufficient reliability, with Cronbach's alpha coefficients ranging from .80 to.89 points for the entire scale.

The validity of the Perceived Stress Questionnaire was also established through expert consultation, yielding a correlation coefficient of 0.76 ([Cohen et al., 1983](#_ENREF_5)). This questionnaire consists of 14 questionsscored on a 5-point Likert scale, where responses range from "never" to "always," with scores assigned from 0 to 4. Specific questions are scored in reverse (4, 5, 6, 7, 9, 10, and 13). A total score below 28 indicates low perceived stress, while a score of 28 or above categorizes the respondent as experiencing high perceived stress.
